# A new plasmid-based microRNA inhibitor system that inhibits microRNA families in transgenic mice and cells: a potential new therapeutic reagent

**DOI:** 10.1038/gt.2016.22

**Published:** 2016-04-07

**Authors:** H Cao, W Yu, X Li, J Wang, S Gao, N E Holton, S Eliason, T Sharp, B A Amendt

**Affiliations:** 1 Institute of Biosciences and Technology, Texas A&M Health Science Center, Houston, TX, USA; 2 Department of Anatomy and Cell Biology, Carver College of Medicine, University of Iowa, Iowa City, IA, USA; 3 Dows Institute of Dental Research, College of Dentistry, The University of Iowa, Iowa City, IA, USA; 4 Craniofacial Anomalies Research Center, Carver College of Medicine, University of Iowa, Iowa City, IA, USA

## Abstract

Current tools for the inhibition of microRNA (miR) function are limited to modified antisense oligonucleotides, sponges and decoy RNA molecules and none have been used to understand miR function during development. CRISPR/Cas-mediated deletion of miR sequences within the genome requires multiple chromosomal deletions to remove all functional miR family members because of duplications. Here, we report a novel plasmid-based miR inhibitor system (PMIS) that expresses a new RNA molecule, which inhibits miR family members in cells and mice. The PMIS engineered RNA optimal secondary structure, flanking sequences and specific antisense miR oligonucleotide sequence bind the miR in a stable complex to inhibit miR activity. In cells, one PMIS can effectively inhibit miR family members that share the same seed sequence. The PMIS shows no off-target effects or toxicity and is highly specific for miRs sharing identical seed sequences. Transgenic mice expressing both *PMIS-miR-17-18* and *PMIS-miR-19-92* show similar phenotypes of *miR-17-92*-knockout mice. Interestingly, mice only expressing *PMIS-miR-17-18* have developmental defects distinct from mice only expressing *PMIS-miR-19-92* demonstrating usefulness of the PMIS system to dissect different functions of miRs within clusters. Different PMIS miR inhibitors can be linked together to knock down multiple miRs expressed from different chromosomes. Inhibition of the *miR-17-92*, *miR-106a-363* and *miR-106b-25* clusters reveals new mechanisms and developmental defects for these miRs. We report a new tool to dissect the role of miRs in development without genome editing, inhibit miR function in cells and as a potential new therapeutic reagent.

## Introduction

MicroRNAs (miRs) are short noncoding RNA molecules, ~22 nucleotides (nts) long, that regulate messenger RNA (mRNA) transcripts post-transcriptionally through binding to complementary sequences on target mRNA.^1, 2, 3, 4^ The human genome may contain over 1500 miR species (miRBase, release 18) and it has been estimated that more than half of protein coding genes could be regulated by miRs.^5, 6^ Since the first discovery in 1993, miRs have been shown to be involved in the regulation of a broad range of biological processes and the malfunction of miRs are associated with many human diseases.^7, 8, 9, 10, 11, 12, 13, 14, 15, 16^

Given the importance of miRs during different biological processes, tools for repression of miR function will not only be useful for research but also have therapeutic potential. Currently, one method to attenuate miR activity is administration of antisense oligos into cells that compete for binding with endogenous targets. These include anti-miR antisense oligonucleotides, which has some or all of the ribonucleotides modified, such as 2′-O-methylated RNA,^17, 18, 19^ locked nucleic acids or 2′-methoxyethylated RNA.^20, 21^ Other modifications of these AMOs include phosphorothioate substitutions, addition of flanking sequences and lipids.^22, 23^ These modifications can increase their affinity towards miR sequences and protect the oligos from processing by cellular nucleases. Other chemically modified antisense oligonucleotides with a 2′-fluoro/2′-methoxyethyl modified antisense oligonucleotide motif improved *in vivo* inhibition of miR activity.^24^ A limitation of these miR inhibitors resides in their inability to be retained in the tissues after cell division and they must be reapplied to maintain their effectiveness. To address these limitations and promote long-term repression of specific miRs, several plasmid and/or viral vectors expressing antagomirs, sponges, eraser and Tough Decoy (TuD) RNA molecules have been reported.^25, 26, 27, 28^ This system and others can inhibit miR activity without degradation of the miR.^24, 29^

Here, we report a new plasmid-based miR inhibitory system (PMIS) based on hairpin structures that specifically bind miR transcripts. The addition of short hairpin structure flanking the antisense sequence greatly increased its inhibitory activity. These structures may coordinate physical interactions with proteins that bring the antisense sequence close to the miR and markedly facilitate miR binding. The PMIS expresses anti-miR antisense sequence flanked by hairpin structures and contain features including AU-rich flanking sequences and the plasmid may be transiently or constitutively expressed depending on the vector or integration. The PMIS effectively and specifically knocks down specific miRs in cells based on the anti-miR antisense sequence. More impressively, the PMIS inhibits miR expression in mice and can be used to dissect the function of miRs within clusters. The PMIS effectively inhibits miR expression in cells and tissues and is a potential therapeutic reagent for cancer and other diseases.

## Results

### Design and optimization of the PMIS

**Figure 1:**
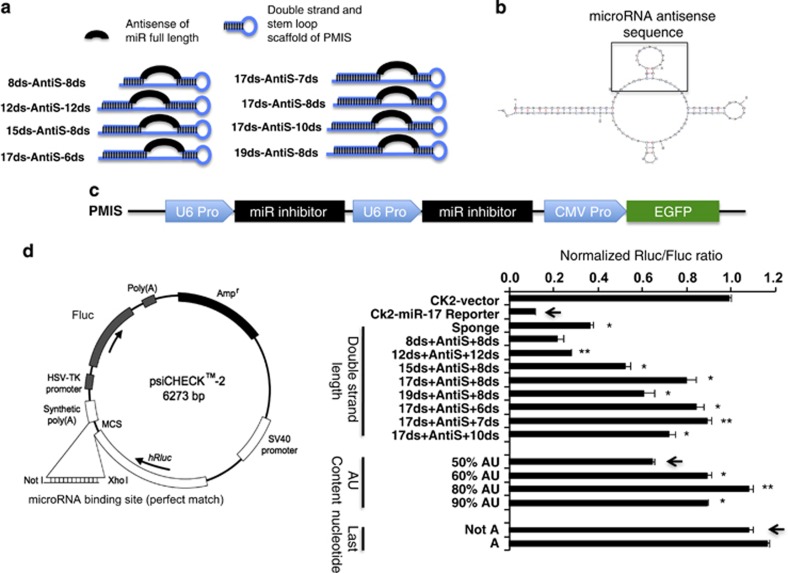
Optimization of miR inhibitor efficiency. (a) miR inhibitor designs. Black line indicates the specific inhibitor sequence that is complementary to the full-length miR. Blue line indicates the double-stranded (ds) and stem loop RNA. (**b**) Predication of RNA secondary structure of the final inhibitor design by RNA mfold. The miR antisense sequence is boxed. (**c**) Structure of the vector construct used to transduce cells, either one or multiple miR inhibitors can be cloned into the vector. (**d**) Effects of secondary structure length, local AU content and last A ribonucleotide on miR inhibitor efficiency. The psiCHECK2 vector (Ck2) does not contain a *miR-17* binding site after Rluc. The Ch2-miR-17 reporter contains a single *miR-17* binding site cloned after Rluc. All co-transfections with inhibitors use the Ck2-miR-17 reporter. Normalized Rluc to Fluc ratio of miR reporter vector without *miR-17* binding site was set as 100%. miR inhibitor designs were co-transfected with miR reporter containing a *miR-17* binding site into HEK 293FT cells, which endogenously express *miR-17*. Rluc and Fluc activity was measured 48 h after transfection. The sponge plasmid has six tandem *miR-17* binding sites. *T*-test was performed against the sample, marked by an arrow. *P<0.05 and **P<0.01. CMV, cytomegalovirus; EGFP, enhanced green fluorescent protein; HSV, herpes simplex virus.

The PMIS design started with an anti-miR oligodeoxyribonucleotide-based approach that expressed a nucleic acid sequence that is antisense to the miR.^22, 30, 31, 32, 33, 34^ The antagomirs used in our study bind to the complete miR sequence including the seed region and flanking sequences to enhance the specificity and binding affinity of the antagomirs. The antagomir sequence was ligated to a custom-designed ~120 nt RNA secondary structure that facilitates its function, stability and processing. The construct is expressed using the U6 Pol III promoter, which does not produce as many transcripts as Pol II promoter (cytomegalovirus) activation. Each nucleotide of the 120-nt backbone RNA structure was selected for its effect on the specificity and mature miR inhibition activity. The initial design began with an 8 nt double-stranded (ds) sequence flanking each end of the antagomir (stem and stem loop; in blue), 8ds-antiS-8ds (Figure 1a). Multiple constructs were designed with different lengths of each double-stranded region and eight of these are shown and the complete construct with a box highlighting the antagomir is shown (Figures 1a and b). The PMIS-miR inhibitors have a U6 promoter followed by a miR inhibitor, and it can link with a second U6 promoter followed by different miR inhibitor (Figure 1c).

Several RNA elements, including double-stranded regions, local AU content and the last nucleotide of the miR binding site influence miR recognition of its endogenous targets.^35^ We reasoned that including these elements might enhance miR binding to the inhibitor (antagomir) that only has a single miR binding site. A series of *miR-17* antisense sequences (inhibitor) were constructed containing different flanking regions. To test the efficiency of these different designs, the inhibitors were co-transfected with a *miR-17* reporter that has the *miR-17* binding site cloned after the luciferase gene into HEK 293FT cells (Figure 1d). The *miR-17* reporter has a perfect *miR-17* binding sequence used in previous reports.^27^ *miR-17* was used to test the inhibitor design, as *miR-17* is one of highest expressed miRs. A miR sponge plasmid that has six tandem *miR-17* binding sites was used as a control. These data demonstrate that the optimal miR inhibitor has a 17 nt double-stranded RNA region at the 5′-end and 7-nt double-stranded sequence at the 3′-end (17ds+antiS+7ds), local AU content is 80% and the last nucleotide is an A (Figure 1d). This miR inhibitor recovers over 90% of the inhibition of luciferase activity by endogenous *miR-17* (Figure 1d).

**Figure 2:**
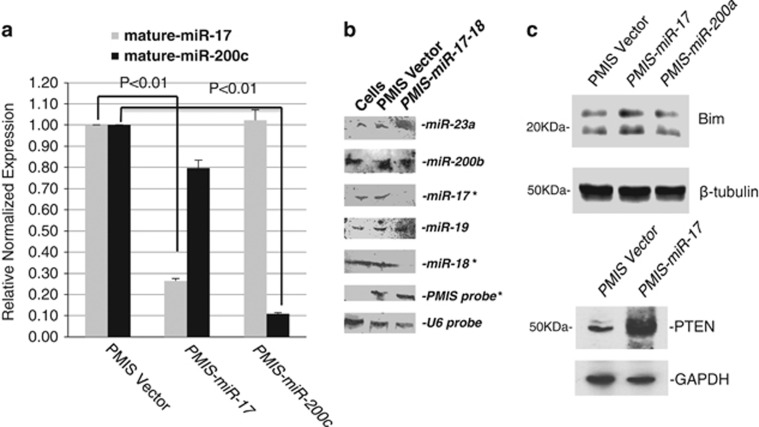
Efficiency of the PMIS. (**a**) PMIS-miR inhibitors reduce specific miR expression. RT-PCR of mature endogenous *miR-17* and *miR-200c* from 293 cells transfected with the indicated PMIS inhibitor. (**b**) Northern blot of mature endogenous *miRs* and the PMIS construct from 293 cells transfected with PMIS vector or *PMIS-miR-17-18*. *U6* RNA is shown as a loading control. (**c**) Western blot of Bim, a known target of *miR-17* from cells transfected with the PMIS vector, *PMIS-miR-17* or *PMIS-miR-200a*. In addition, a western blot of PTEN, identified as a target of *miR-17*. β-Tubulin and GAPDH (glyceraldehyde 3-phosphate dehydrogenase) are shown as loading controls.

The *miR-17* inhibitor (*PMIS-miR-17*) reduced endogenous mature *miR-17* levels to ~25% in 293 cells, whereas the *miR-200c* inhibitor (*PMIS-miR-200c*) does not change the level of *miR-17* (Figure 2a). *PMIS-miR-200c* inhibits *miR-200c* to ~90% but does not affect *miR-17* levels (Figure 2a). The reduction of miR levels using a sponge miR inhibitor was previously reported to be an artifact of the detection method;^27^ therefore, we performed Northern blots for *miR* expression after transduction of the *PMIS-miR-17-18* construct in 293 cells (Figure 2b). These results show that *miR-17* and *miR-18* levels were reduced by *PMIS-miR-17-18* and this is consistent with other studies demonstrating miR degradation by inhibitors.^36, 37^ As controls, the levels of m*iR-23a*, *miR-200b* and *miR-19* were not affected by *PMIS-miR-17-18*. We also wanted to determine if the PMIS vector and/or *PMIS-miR-17-18* construct were degraded or cleaved in the cells; however, both were expressed and not cleaved by Dicer (Figure 2b). However, it is also likely that the PMIS system can inhibit miR activity by sequestration and slow degradation of the miR as we detect low levels of miR expression.^24, 29^ *Bim* expression, a proapoptotic gene involved in B-cell development and a known target of *miR-17*,^38^ was elevated in cells transfected with *PMIS-miR-17* compared with cells with empty vector and *PMIS-miR-200a* (Figure 2c). Furthermore, Bim transcripts levels were increased ~3-fold in *PMIS-miR-17* cells (data not shown). PTEN (phosphatase and tensin homolog) was identified in a screen for genes regulated by *miR-17* and we show that inhibition of *miR-17* by *PMIS-miR-17-18* increased PTEN protein expression (Figure 2c).

### Specificity of the miR inhibitor

**Figure 3:**
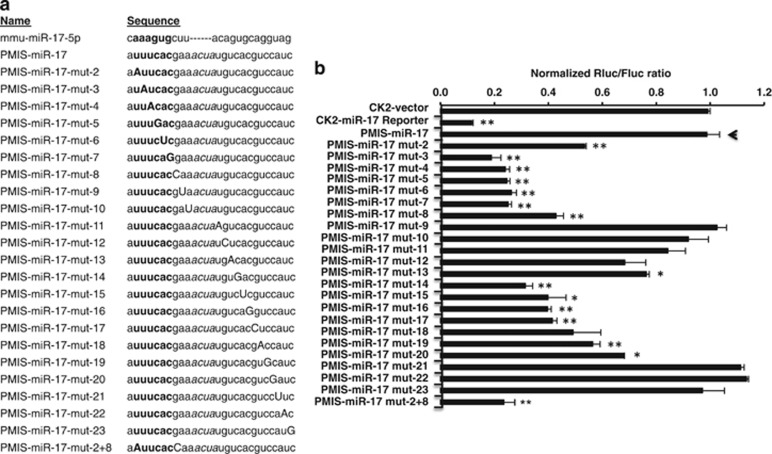
Specificity of miR inhibitors. (**a**) Sequence of *miR-17* inhibitor and specific mutations to test function. The numbers indicate the mutated ribonucleotide (bold and in caps) and the AUCA sequence in the middle of the inhibitor sequence eliminates a perfect match.^27^ The seed sequence of the mature *miR-17-5p* is in bold as is the antisense sequence to the seed sequence in *PMIS-miR-17*. (**b**) Ribonucleotide mutation effects on miR inhibitor efficiency. Normalized Rluc to Fluc ratio of miR reporter vector without *miR-17* binding site was set as 100%. The *PMIS-miR-17* inhibitor mutants were co-transfected with miR reporter vector containing a *miR-17* binding site into HEK 293FT cells. Rluc and Fluc activity was measured 48 h after transfection. *T*-test was performed against the sample, marked by arrow. *P<0.05 and **P<0.01.

Specific nucleotides were mutated in *PMIS-miR-17* (Figure 3a) and tested for function using the dual luciferase assay as in Figure 1d. As expected, a single mutation of the inhibitor corresponding to the seed sequence (nts 2–8, mut-2 to 8) of *miR-17* almost completely eliminated its effects (Figures 3a and b). Another region important for miR inhibitor function corresponds to nts 12–20 (mut-12 to 20) (Figures 3a and b). Other nucleotide changes do not significantly impair miR inhibitor function. As expected, changing two nucleotides corresponding to the seed region (mut-2+8) has additional additive effects (Figures 3a and b).

### *miR-200* family knockdown by a single miR inhibitor plasmid

**Figure 4:**
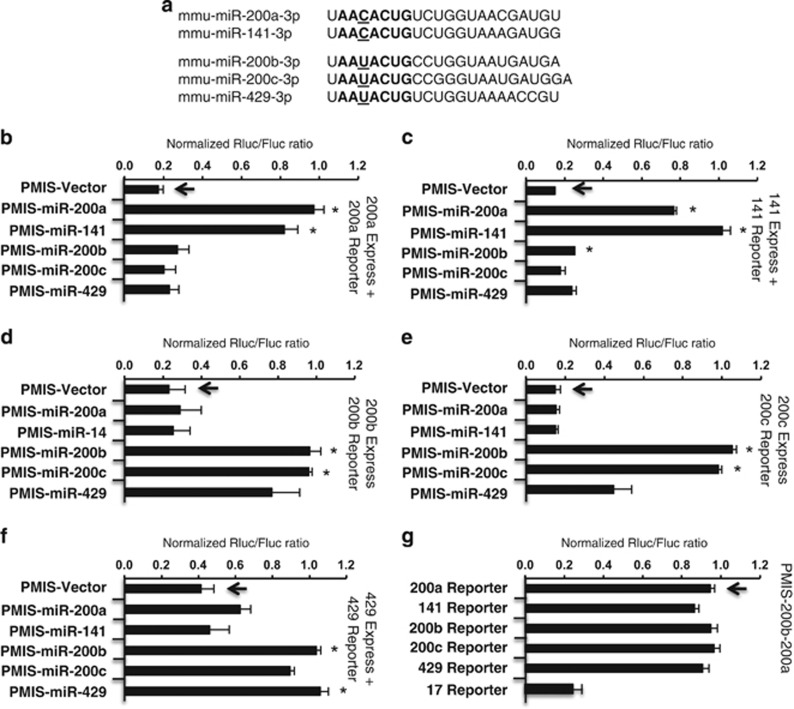
Knockdown of the *miR-200* family with a single plasmid. (**a**) Sequence of mature *miR-200* family members. The underlined nucleotide indicates the different ribonucleotide between two subfamilies in the seed region (bold). (**b**) Inhibition of *miR-200a-3p* with different PMIS-miR inhibitors. PMIS empty vector or various PMIS-miR inhibitors were co-transfected with *miR-200a* expression plasmid and miR reporter vector containing a *miR-200a* binding site into HEK 293FT cell. Rluc and Fluc activity was measured 48 h after transfection. (**c**) Inhibition of *miR-141-3p* with different PMIS-miR inhibitors. (**d**) Inhibition of *miR-200b-3p* with different PMIS-miR inhibitors. (**e**) Inhibition of *miR-200c-3p* with different PMIS-miR inhibitors. (**f**) Inhibition of *miR-429-3p* with different PMIS-miR inhibitors. (**g**) A single plasmid expressing both *PMIS-miR-200a-200b* inhibits all five members of the *miR-200* family. The *miR-17* reporter was used as a control. *T*-test was performed against the sample, marked by arrow. *P<0.05.

Because one miR inhibitor can potentially inhibit different members of the same miR family, we asked if a PMIS construct containing two inhibitors targeting an miR family could inhibit the complete miR family. To test this, cells were transfected with the miR reporter luciferase construct, the associated miR that binds to the target in the luciferase construct and the miR inhibitors or empty vector. The *miR-200* family has two subfamilies, *miR-200a-3p/141-3p* and *miR-200b-3p/200c-3p/429-3p* (Figure 4a). These two subfamilies have one nucleotide difference (underlined) in their seed regions. PMIS constructs for *miR-200a* or *-141* (*PMIS-miR-200a* or *PMIS-miR-141*) inhibited the *miR-200a/141* subfamily but not the *miR-200b/200c/429* subfamily (Figures 4b and c). This result is consistent with other previous data that an miR inhibitor could inhibit several miRs that share the same seed sequence.^39, 40^ PMIS for *miR-200b*, *-200c* or *-429* inhibited the *miR-200b/200c/429* subfamily efficiently but not the *miR-200a/141* subfamily (Figures 4d–f). However, the inhibitor to *miR-429* was not as effective at knocking down *miR-200c* expression compared with inhibitors *200a* and *200b*, due to flanking sequence divergence of *miR-429* (and the inhibitor). To inhibit the *miR-200* family, *PMIS-miR-200b-200a* was constructed and contains a U6 promoter followed by an *miR-200b* inhibitor, a second U6 promoter followed by *miR-200a* inhibitor (Figure 1c). This construct allows for the expression of multiple miR inhibitors from one plasmid to inhibit the entire *miR-200* family (Figure 4g). The *miR-17* reporter was used as a control to show that *PMIS-miR-200b-200a* did not affect endogenous *miR-17* activity.

Overexpression of *miR-200* promotes a mesenchymal-to-epithelial transition in MDCK-Pez cells and conversely inhibition of *miR-200* causes EMT in MDCK cells.^41^ To test if the *PMIS-miR-200* inhibits *miR-200* function *in vitro* and promotes EMT, a stable MDCK cell line was made that expresses *PMIS-miR-200a-200b*. Immunofluorescence of the epithelial marker E-cadherin confirmed that MDCK cells underwent EMT after expression of *PMIS-miR-200a-200b* (data not shown).

### The PMIS inhibits noncanonical miRs and siRNA

**Figure 5:**
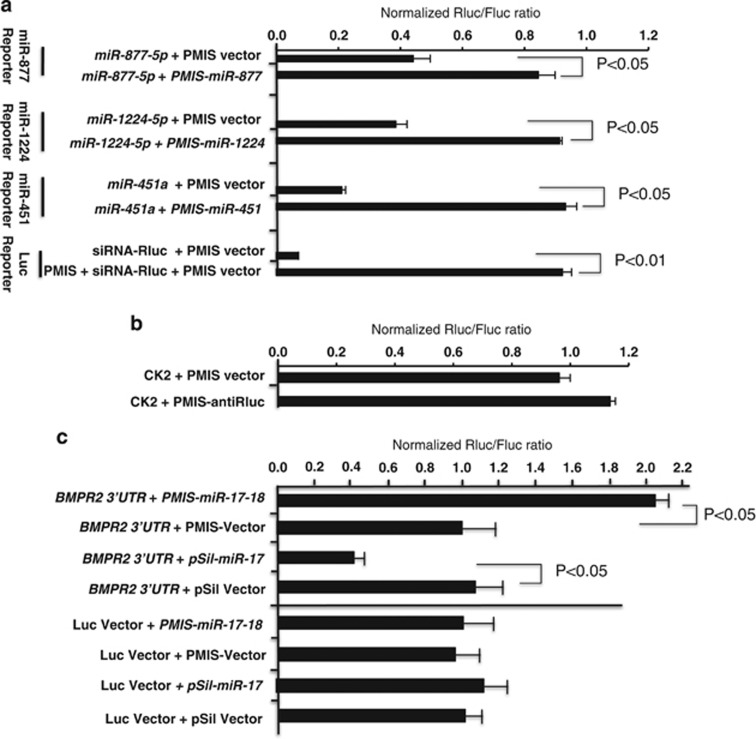
Efficiency of PMIS-miR inhibitor targeting noncanonical miRs. (**a**) Efficiency of PMIS-miR inhibitors targeting intronic miRs that bypass Drosha processing *miR-877-5p* and *miR-1224-5p*, Dicer-independent *miR-451a* and *in vitro* transcribed *siRNA*. PMIS-miR inhibitor targeting Rluc mRNA served as a control. Rluc and Fluc were assayed as in previous figures. (**b**) As an additional control, *PMIS-anti-Rluc* had no effect on luciferase expression and activity. (**c**) The *BMPR2 3′UTR* was cloned into the luciferase vector and co-transfected with *PMIS-miR-17-18*, vector only or *pSil-miR-17* (overexpression) in 293 cells.

In addition to the canonical miR biogenesis pathway, there are other types of miRs that do not require Drosha or Dicer to process them for functionality. To test whether the PMIS inhibits noncanonical miRs, inhibitors were made to two miRs that do not require Drosha (*miR-877-5p* and *miR-1224-5p*) and one that does not require Dicer (*miR-451a*). The miR inhibitors inhibited both miRs (Figure 5a). small interfering RNAs (siRNAs) are different from miRs in that they do not require Drosha and Dicer processing for function. However, both siRNAs and miRs load into a functional RNA-induced silencing complex (RISC). To test whether the PMIS also works on siRNAs, we designed an siRNA to luciferase mRNA. The PMIS to this siRNA efficiently blocks siRNA function (Figure 5a). As a negative control, this PMIS does not knock down luciferase activity by itself (Figure 5b). Thus, the PMIS works specifically on all miRs and siRNAs.

The *BMPR2* gene was identified as a target of *miR-17* in *PMIS-miR-17-18* bioinformatics analyses of transgenic mouse tissues. To demonstrate that *PMIS-miR-17-18* can also inhibit endogenous *miR-17* from repressing *BMPR2* expression, the *BMPR2 3′UTR* (untranslated region) was cloned into the luciferase vector and co-transfected with *miR-17* or vector only and *PMIS-miR-17-18* or vector only. *PMIS-miR-17-18* caused a twofold increase in luciferase activity, whereas *miR-17* overexpression decreased luciferase activity 60% compared with controls (Figure 5c).

### miR inhibitor associates with Dicer and argonaute

**Figure 6:**
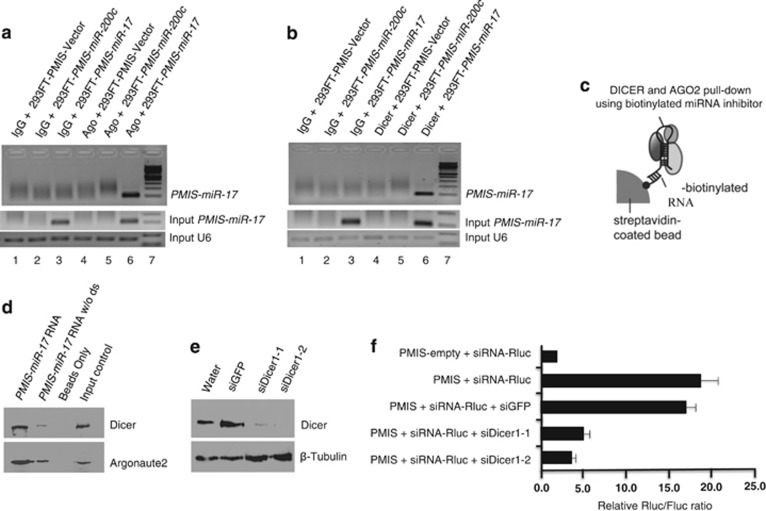
miR inhibitor interaction with RISC. (**a**) Immunoprecipitation (IP) of miR inhibitor using AGO2 antibody. PCR primers were used to detect *PMIS-miR-17* after pull down, and *U6* was used as loading control. Immunoglobulin G antisera was used as a control and *PMIS-miR-200c* transfected as a PCR control. (**b**) IP of miR inhibitor using a Dicer antibody. Controls were as in panel a. (**c**) Schematic of the Dicer and Ago2 pull-down assay. (**d**) Pull down of AGO2 and Dicer with biotinylated *PMIS-miR-17*. Biotinylated *PMIS-miR-17* RNA or *PMIS-miR-17* RNA without the double-stranded regions were incubated with 293 cell extracts, washed, denatured and analyzed by polyacrylamide gel electrophoresis (PAGE). Western blot was performed using Dicer and AGO antibodies. Proteins were detected using ECL reagents (GE Healthcare). (**e**) Western blot of Dicer after knockdown of Dicer with siRNA. Cell lysates (15 μg) were resolved by PAGE after transfection with siGFP, siDicer 1-1 and siDicer 1-2 (two different siRNA constructs) or water as a control. Western blot was performed using Dicer antibody and β-tubulin antibody as a loading control. (**f**) Knockdown of Dicer impaired miR inhibitor function. Rluc vector and *in vitro* transcribed siRNA targeting Rluc was co-transfected with miR inhibitor to siRNA-Rluc and *in vitro* transcribed siRNA targeting GFP or two different sites of the Dicer transcript. Rluc and Fluc activity was measured 48 h after transfection.

To determine whether the PMIS constructs were bound by argonaute (AGO), 293 cells were transduced with PMIS vector only, and *PMIS-miR-200c* or *PMIS-miR-17* were subjected to immunoprecipitation (IP) assays using either immunoglobulin G (control) or AGO antibodies. After IP, the complexes were washed and denatured and PCR was performed using primers for *PMIS-miR-17*. The complexes containing endogenous *miR-17* and *PMIS-miR-17* were pulled down by the AGO antibody (Figure 6a, lane 6). A similar IP used the Dicer antibody to pull down the inhibitor complex and Dicer was associated with *PMIS-miR-17* (Figure 6b, lane 6). In addition, proteins were pulled down that associated with the miR inhibitor using biotin *in vitro* transcribed RNA and incubated with cell lysates and probed for Dicer and AGO binding by western blot (Figure 6c). The PMIS associates with Dicer and AGO and this association decreased when the miR inhibitor lost its double-stranded RNA structure (Figure 6d). The PMIS doubled-stranded RNA stem structure (5′ end) appears to bind Dicer; however, because of its short length 18 nts, Dicer does not cleave it as Dicer cleaves 22–24 nt long double-stranded RNA sequences. This lack of PMIS cleavage was also shown by Northern blot in Figure 2. Furthermore, Dicer recognizes the PMIS double-stranded stem structure but not the PMIS-miR interaction, due to the 4 nts mismatch in the antisense oligo design.

To test whether Dicer was required for PMIS function, siRNA was used to knock down Dicer (Figure 6e). The PMIS inhibits siRNA, which targets the luciferase gene (*Rluc*) and siGFP has no effect, used as a control (Figure 6f). However, after knocking down Dicer with siRNA the miR inhibitor to siRNA-Rluc has no effect on inhibiting the activity of the siRNA to Rluc (Figure 6f).

### The PMIS is relatively stable in cells

**Figure 7:**
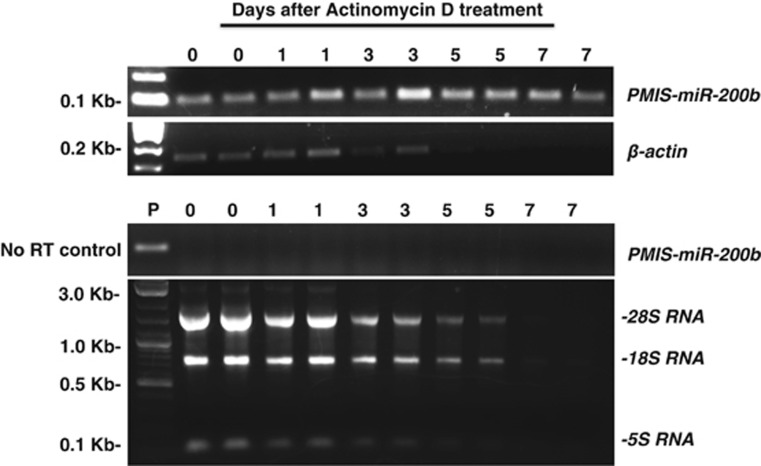
PMIS-miR inhibitors are relatively stable in the cell. Cells stably expressing *PMIS-miR-200b* were seeded at identical concentrations into six- well plates and subjected to actinomycin D treatment for 7 days. After 1, 3, 5 and 7 days, cells were harvested, total RNA harvested and *PMIS-miR-200b* was analyzed by RT-PCR (top panel). β-Actin was used as a control. The bottom panel shows the 28 S, 18 S and 5 S RNAs as controls. Another control contained the isolated total RNA without reverse transcription (no RT control) used in the PCR reaction to detect possible PMIS DNA; however, no PMIS DNA was detected in the samples.

To determine the relative stability of the miR inhibitor, cells were transduced with lentivirus expressing *PMIS-miR-200b*. The cells were seeded at identical densities into 6-well plates, and after 24 h, they were subjected to actinomycin D (5 ng/ml) treatment for the indicated time (days). The PMIS showed no decrease in transcript stability after 7 days of treatment, compared with β-actin (Figure 7, top panel). However, 28 S, 18 S and 5 S RNAs were all degraded after 5 days of treatment with actinomycin D (Figure 7, bottom panel). Because the average miR half-life is ~5 days, the PMIS appears to be more stable than miRs, allowing them to remain functional in the cell.^42^ The actinomycin D-treated cells were dead or dying after 4–5 days and RNA analyzed after 4 days was from dead or dying cells left on the plates. Furthermore, because a single miR can recycle to process multiple mRNA transcripts, the PMIS stability may facilitate longer interactions with target miRs and provide more efficient inhibition of their activity.^37^

### Analyses of gene regulation by *PMIS-miR-17*

**Figure 8:**
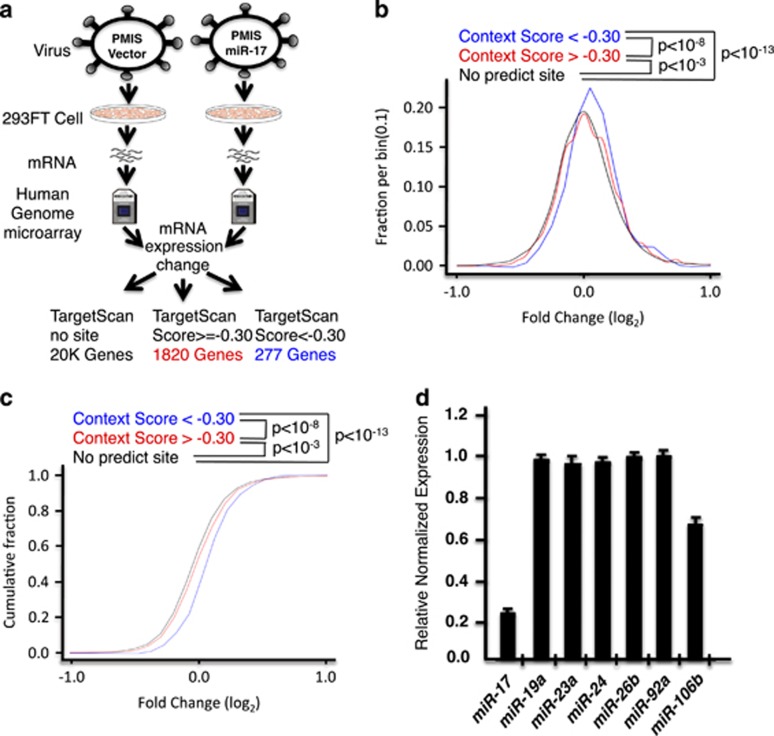
Genome-wide analyses of *PMIS-miR-17* function. (**a**) Experiment design for the whole-genome-wide analysis of mRNA change after transduction with *PMIS-miR-17*. (**b** and **c**) Changes in abundance of mRNAs in *PMIS-miR-17*-expressing 293 cells were monitored by microarrays. TargetScan was used to predict *miR-17* targets. We further separate *miR-17* targets into two groups, one with context+ score <−0.30 and the other with context+ score >−0.30. Distributions of changes (0.1 unit bins) for mRNA UTRs containing no site in black line, site with score >−0.30 in red line and site with score <−0.30 in blue line. Upregulation of mRNAs with context+ score <−0.30 was significantly more than that from mRNAs with no site (*P*<10^−13^, one-sided K–S test). Comparisons between mRNAs with context+ score <−0.30 versus context+ score >−0.30, context+ score >−0.30 versus no site were also significant (*P*<10^−8^, *P*<10^−3^, respectively). (**d**) The 293 cells were transduced with *PMIS-miR-17*, total RNA harvested and randomly selected miR expression was analyzed by RT-PCR using Taqman probes (*N*=3).

To evaluate PMIS efficacy and specificity in a systematic analysis, we profiled whole-genome mRNA expression changes after transduction with *PMIS-miR-17* in 293 cells. A schematic of the experimental design is shown (Figure 8a). The *miR-17* targets were separated into two groups based on TargetScan prediction score, one with a context+ score <−0.30 and the other with a context+ score >−0.30. Distributions of changes (0.1 unit bins) for mRNA UTRs containing no site (black line), site with a score >−0.30 (red line) and site with a score <−0.30 (blue line) shows mRNA targets of *miR-17* are upregulated in *PMIS-miR-17* transfected cells (Figures 8b and c). Upregulation of mRNAs with context+ score <−0.30 was significantly more than that from mRNAs with no site (*P*<10^−13^, one-sided Kolmogorov–Smirnov (K–S) test). Comparisons between mRNAs with context+ score <−0.30 versus context+ score >−0.30; context+ score >−0.30 versus no site were also significant (*P*<10^−8^ and *P*<10^−3^, respectively) (Figures 8b and c). These results demonstrate that *PMIS-miR-17* specifically and effectively inhibit *miR-17*, which in turn results in the increase in transcripts targeted by *miR-17*.

To determine if *PMIS-miR-17* inhibited other miRs in cells, 293 cells were transduced with the lentiviral *PMIS-miR-17* and randomly selected miRs were profiled by real-time PCR (q-PCR) (Taqman probes; Applied Biosystems, Waltham, MA, USA). *PMIS-miR-17* was specific for the inhibition of *miR-17-5p* and *miR-106b-5p* (Figure 8d).

### PMIS inhibits miRs during mouse development

**Figure 9:**
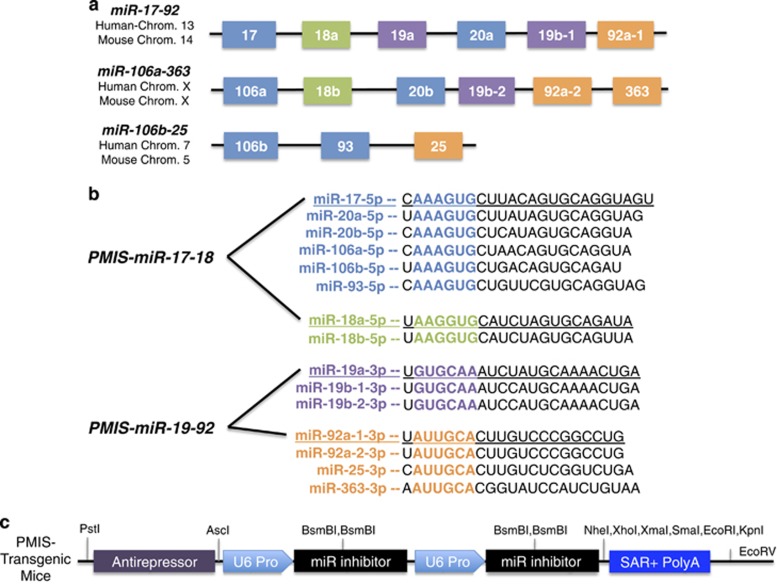
Schematic of the *miR-17-92*, *miR-106a-363* and *miR-106b-25* clusters. (**a**) The location and organization of the miR clusters are shown in the top panel. (**b**) The middle panel shows the sequence similarities and differences between the miRs. *PMIS-miR-17* and *PMIS-miR-18* were derived from *miR-17-5p* and *miR-18a-5p*, respectively. *PMIS-miR-19* and *PMIS-miR-92* were derived from *miR-19a-3p* and *miR-92a-1-3p*, respectively. (**c**) The structure of the construct used for pronuclear injection is shown at the bottom of figure.

The PMIS was designed as a pronuclear-injected construct to create transgenic mice that stably express the miR inhibitors. Because the *miR-17-92* intragenic cluster has been conventionally deleted, we compared our *PMIS-miR-17-18-19-92* transgenic mice phenotype to the published *miR-17-92* knockout.^38, 43, 44, 45^ However, the conventional *miR-17-92* knockout does not delete the other miR family clusters, *miR-106a-363* and *miR-106b-25* (Figure 9a). We made two transgenic mice to inhibit all three clusters. Because the *miR-17-92*^*null*^ mice are perinatal lethal, two PMIS constructs *PMIS-miR-17-18* and *PMIS-miR-19-92* were pronuclear injected to make transgenic (TG) mice (Figure 9b, underlined sequence). These four miR inhibitors target the four seed regions shared among the miR families (Figures 9a and b). Each PMIS transgene was designed with an antirepressor element upstream of the mouse U6 promoter followed by an miR inhibitor, a second U6 promoter followed by different miR inhibitor, a scaffold-attached region (SAR) and polyA site (Figure 9c).

**Figure 10:**
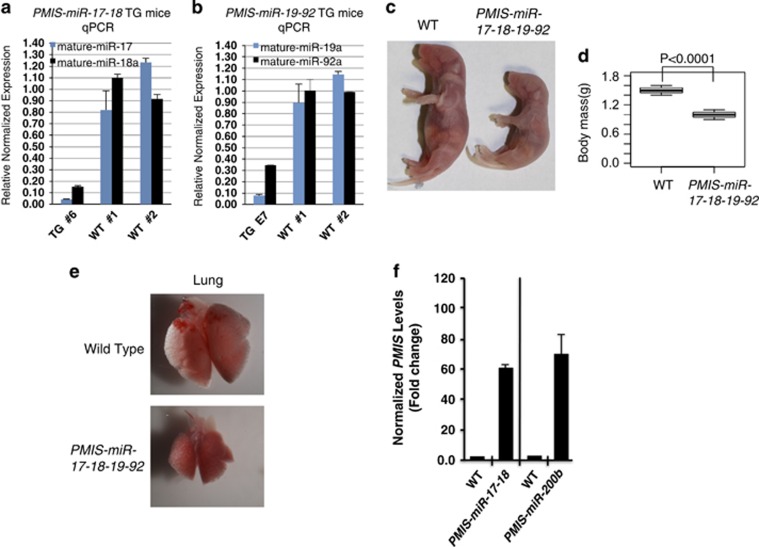
*PMIS-miR-17-18-19-92* function in mice. (**a**) RT-PCR of endogenous mature *miR-17* and *miR-18a* in *PMIS-miR-17-18* transgenic mice and control littermates. Taqman probes were used to analyze miR expression from mouse tails. (**b**) RT-PCR of endogenous mature *miR-19a* and *miR-92a* in *PMIS-miR-19-92* transgenic mice and control littermates. Taqman probes were used to analyze miR expression from mouse tails. (**c**) *PMIS-miR-17-18* and *PMIS-miR-19-92* mice were mated to produce the *PMIS-miR-17-18-19-92* embryos that are perinatally lethal. The E18.5 *PMIS-miR-17-18-19-92* embryos are smaller than WT embryos. (**d**) The weight of the *PMIS-miR-17-18-19-92* embryos are less than their littermates. (**e**) The *PMIS-miR-17-18-19-92* embryos have small lungs. (**f**) qPCR of *PMIS-miR-17-18* and *PMIS-miR-200b* levels in transgenic mice expressing these constructs. Total RNA was isolated from E18.5 mandible tissue.

Multiple founders were generated and *PMIS-miR-17-18* no. 6 demonstrated 95% knockdown of *miR-17* and 86% knockdown of *miR-18a* by RT-PCR (Figure 10a). Multiple founders for *PMIS-miR-19-92* showed decreased miR expression, *PMIS-miR-19-92* no. 7 inhibited *miR-19a* at 95% and *miR-92a* at ~65% (Figure 10b). Founders *PMIS-miR-17-18 no. 6* and *PMIS-miR-19-92 no. 7* were established and these mice were crossed to generate the *PMIS-miR-17-18-19-92* TG mice. The *PMIS-miR-17-18* and *PMIS-miR-19-92* mice are viable; however, ~25% have craniofacial defects that cause neonatal lethality. The *PMIS-miR-17-18-19-92* mice are perinatal lethal similar to the *miR-17-92*^*null*^ mice. *PMIS-miR-17-18-19-92* embryos (E18.5) are smaller than wild-type (WT) embryos with reduced body weight as reported for the *miR-17-92*^*null*^ mice (Figures 10c and d).^38, 44^ These mice also have small lungs (Figure 10e). Quantitative PCR of PMIS expression in two different PMIS transgenic embryos show high levels of *PMIS-miR-17-18* and *PMIS-miR-200b*, respectively (Figure 10f). Because the WT mice do not express the PMIS, the fold change is compared with no expression. In Northern blots showing PMIS expression, the levels of transcripts are similar to miRs (see Figure 2).

### *miR-17-92* expression is inhibited in the *PMIS-miR-17-18-19-92* mice

**Figure 11:**
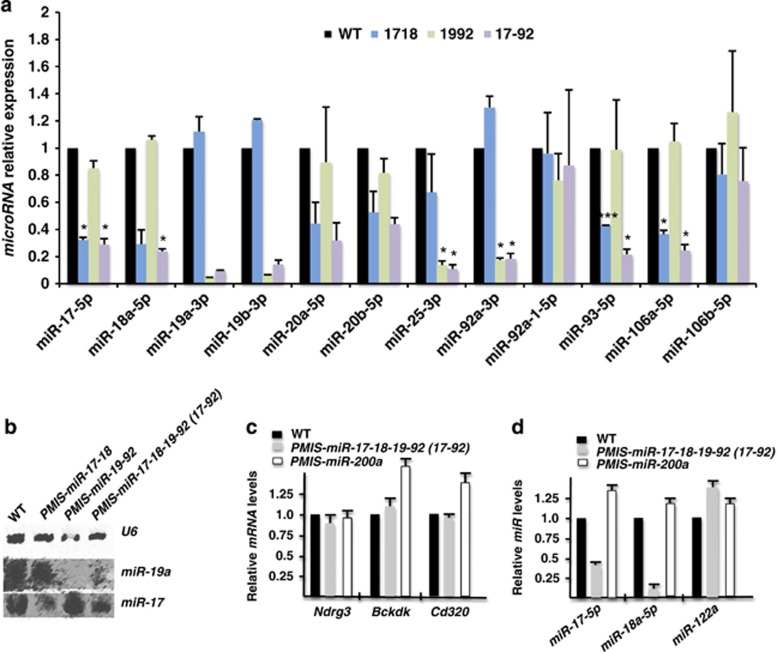
miR expression levels in *PMIS-miR-17-18*, *PMIS-miR-19-92* and *PMIS-miR-17-18-19-92* TG mice. (**a**) Total RNA was harvested from E18.5 mice heads and subjected to miR expression analyses as per the manufacturer's instruction (Qiagen, Hilden, Germany). miR expression is calculated as ΔΔCT after normalization to control primers. All data was compared with WT mice (*N*=2, two different mice and qPCR performed in triplicate for each mouse; *P<0.05). (**b**) Northern blot analyses of *miR-19* and *miR-17* expression in total RNA harvested from E18.5 mouse mandibles. *PMIS-miR-17-18* mice demonstrated reduce *miR-17*; *PMIS-miR-19-92* mice showed reduced *miR-19* and the *PMIS-miR-17-18-19-92* mice had reduced expression of both *miR-17* and *19*. (**c**) qPCR of *Ndrg3*, *Bckdk* and *Cd320* transcripts in total RNA isolated from *PMIS-miR-17-18-19-92, PMIS-miR-200a* or WT E18.5 mouse liver (*N*=2). (**d**) miR expression in RNA isolated from *PMIS-miR-17-18-19-92, PMIS-miR-200a* or WT E18.5 mouse liver.

Mouse tissues from E18.5 *PMIS-miR-17-18*, *PMIS-miR-19-92* and *PMIS-miR-17-18-19-92* TG embryos were analyzed for miR expression. To analyze large miR sets, the Qiagen protocol for miR detection was used to determine miR expression profiles. Specific miR expression associated with *PMIS-miR-17-18* and *PMIS-miR-19-92* mice were decreased and the complete *miR-17-92* family was inhibited in the *PMIS-miR-17-18-19-92* embryos (Figure 11a). Furthermore, *miR-92a-1-5p*, which is not targeted by the inhibitor system, have normal expression level in TG mice. Northern blots of RNA from *PMIS-miR-17-18* embryonic mandibles demonstrate specific reduction of *miR-17*, while *miR-19* was not affected compared with WT (Figure 11b). *miR-19* expression was decreased in *PMIS-miR-19-92* embryos but not in WT or *PMIS-miR-17-18* (Figure 11b). Both *miR-17* and *miR-19* expression levels were decreased in the *PMIS-miR-17-18-19-92* embryos compared with WT (Figure 11b).

To control for toxicity and nonspecific effects of the PMIS inhibitors, total RNA was isolated from P14 mice livers and probed for specific gene transcripts and miR expression levels. *Ndrg3* (N-myc downstream regulated gene 3), *Bckdk* (branched-chain α-ketoacid dehydrongenase kinase) and *Cd320* (Cd320 antigen, putative VLDL receptor), which are also known targets of *miR-122*,^46^ were analyzed for their expression in WT, *PMIS-miR-17-18-19-92* and *PMIS-miR-200a* mice. All three genes, which are associated with liver disease and toxicity were not affected by expression of the PMIS inhibitors (Figure 11c). *miR-122* is associated with liver homeostasis and decreased levels of *miR-122* are seen in hepatocarcinogenesis.^47^ As expected in the *PMIS-miR-17-18-19-92* mice, liver *miR-17* and *miR-18* levels were decreased compared with WT and *PMIS-miR-200a* controls (Figure 11d). However, *miR-122* expression was not decreased (Figure 11d), thus several genes and *miR-122* associated with liver disease were not affected by the expression of the PMIS. While a previous report indicated that shRNA (short hairpin RNA) overexpression could cause cytotoxicity and lethality in mice through saturation of exportin-5 and Ago2, the PMIS mice show no toxicity.^48^ The *PMIS-miR-17-18*, *PMIS-miR-19-92* and *PMIS-miR-200a* mice do not develop cancer or die due to liver toxicity or toxicity problems.

### Craniofacial defects in the *PMIS-miR-17-18-19-92* mice

**Figure 12:**
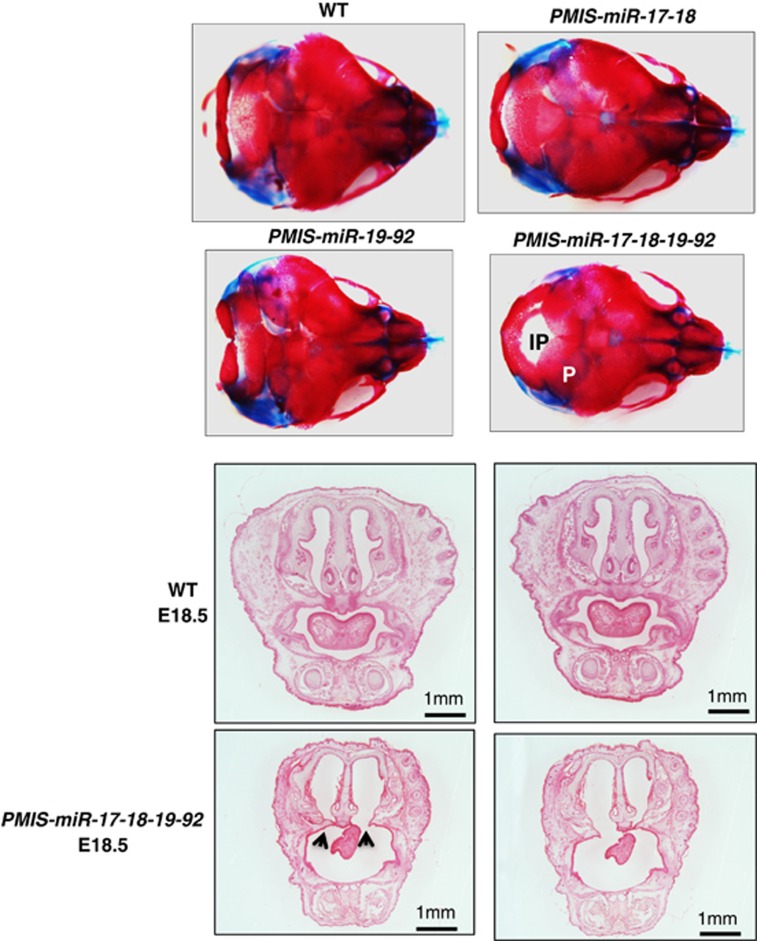
Craniofacial and bone defects in *PMIS-miR-17-18*, *PMIS-miR-19-92* and *PMIS-miR-17-18-19-92* embryos. E18.5 embryos were harvested and heads were stained with Alcian blue and Alizarin red to detect cartilage and bone, respectively. Cross-lines were set to WT size limits and copied onto mutant heads. *PMIS-miR-17-18* embryos have a reduced cranial width compared with WT but normal ossification. *PMIS-miR-19-92* embryos show reduced width and anterior–posterior length but normal ossification. However, *PMIS-miR-17-18-19-92* embryos have reduced anterior–posterior length, reduced cranial width and a delay in ossification of the interparietal (IP) bone. A cleft palate was identified in *PMIS-miR-17-18-19-92* E18.5 embryos, coronal sections, hematoxylin and eosin (H&E) staining (bottom panels, arrows).

The *miR-17-92*^*null*^ mice have defects associated with Feingold syndrome including microcephaly.^44^ Bone and cartilage development was analyzed in E18.5 skulls of *PMIS-miR-17-18* mice, *PMIS-miR-19-92* mice and the combined *PMIS-miR-17-18-19-92* mice compared with WT mice. The *PMIS-miR-17-18-19-92* mice have microcephaly represented as a shortening of the anterior–posterior axis and an overall reduction in size with a decrease in ossification of the parietal (P) and interparietal (IP) bones (Figure 12). Interestingly, the *PMIS-miR-17-18* mice have a normal anterior–posterior axis with normal ossification; however, the skull width is slightly narrower than WT mice (Figure 12). The *PMIS-miR-19-92* mice have a reduced anterior–posterior axis similar to the *PMIS-miR-17-18-19-92* mice and reduced width of the skull in the dorsal view (Figure 12). These two PMIS mice show separation of the effects of the *miR-17-92* cluster where *miR-17* and *-18* control skull width and *miR-19-92* control width and anterior–posterior axis growth, but normal ossification.

The *PMIS-miR-17-18-19-92* mice (E18.5) have a cleft palate as shown for the *miR-17-92*^*null*^ mice.^43^ In the *miR-17-92*^*null*^ the palate shelves elevate and adhere with the nasal septum but do not form the secondary palate and this was observed in the P0 *PMIS-miR-17-18-19-92* mice as well (data not shown). However, several mice also showed a lack of or incomplete palate shelf fusion with the nasal septum (Figure 12).

### Skeletal defects are associated with disrupted *miR-17-92* activity

**Figure 13:**
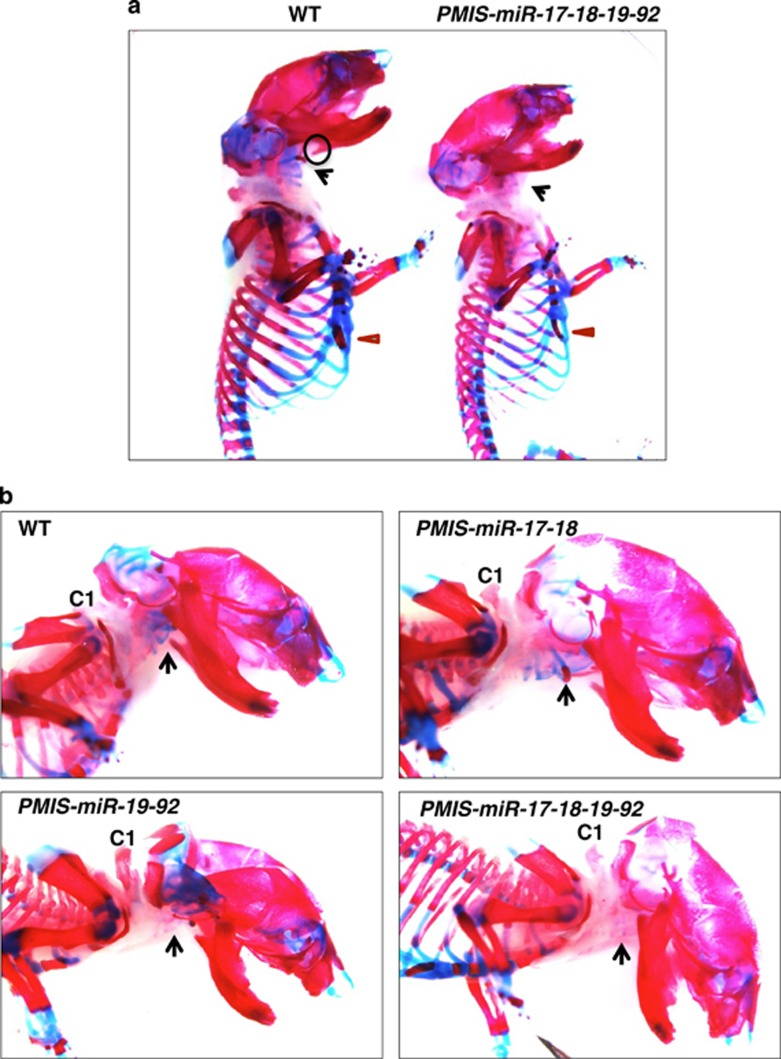
Skeletal defects in the *PMIS-miR-17-18-19-92* embryos. E18.5 embryos were harvested and bodies were stained with Alcian blue and Alizarin red to detect cartilage and bone, respectively. (**a**) *PMIS-miR-17-18-19-92* embryos show delayed overall ossification, lack of thyroid structures (black arrow) and a loss of the processus angularis (circle) and microcephaly. (**b**) The *PMIS-miR-17-18* mice have normal thyroid development and structures, but the first cervical (C1) vertebra is malformed. The *PMIS-miR-19-92* mice have lost the thyroid structures (black arrow), and have a malformed C1 vertebra. The *PMIS-miR-17-18-19-92* mice are defective for multiple cartilages and thyroid structures.

The *PMIS-miR-17-18-19-92* mice (E18.5) were stained with Alcian blue and Alizarin red to detect cartilage and bone, respectively. The *PMIS-miR-17-18-19-92* mice lack the lesser horn of hyoid, greater horn of hyoid, thyroid cartilage and cricoid cartilage (black arrows, Figure 13a). The sternum is smaller (red arrow, Figure 13a) and these mice have fusions of the first three cervical vertebrae as previously shown (data not shown).^44^ Interestingly, the *PMIS-miR-17-18-19-92* mice lack the processus angularis (black circle, Figure 13a).

A unique aspect of the PMIS is its ability to target specific miRs within a cluster. Analyses of *PMIS-miR-17-18* mice show the thyroid cartilages compared with WT (Figure 13b). Although there is dyssymphysis of the first and second cervical vertebrae in this mouse they are not fused as shown for the *miR-17-92*^*null*^ mouse.^44^ The *PMIS-miR-19-92* mice have normal thyroid cartilages with dyssymphysis of the first and second cervical vertebrae (Figure 13b). The *PMIS-miR-17-92* mice have an almost complete loss of thyroid cartilages, loss of the processus angularis and dyssmphysis and fusion of the C1 and C2 vertebrae (Figure 13b). However, *miR-19* and *-92* appear to regulate thyroid and cervical vertebrae development (without fusion of the vertebrae) and mandible and head size (microcephaly) (Figure 13b). This phenotype is repeated in the *PMIS-miR-17-18-19-92* mice; however, these mice have fusion of the cervical vertebrae (not shown) and a loss of the processus angularis and microcephaly (Figure 13b). This is the first report of thyroid deficiency due to *miR-17-92* inhibition in mice. *miR-17-92* inhibition in thyroid cells induces strong growth reduction.^49^

## Discussion

miRs are essential regulators of gene expression and critical for normal embryonic development, tissue morphogenesis, cellular functions such as metabolism and are associated with cancer cell types. However, our knowledge of the full aspects of miR function during development remains fragmentary because of the limited availability of experimental tools to knock down or knock out miRs *in vivo*. Several approaches have been reported to knock down miRs in adult mice but not during embryonic development. One method reported using the Tough Decoy system delivered using recombinant adeno-associated virus injected into adult mice.^50^ The recombinant adeno-associated virus expressing *anti-miR-122* Tough Decoy reduced serum cholesterol by >30% for 25 weeks in mice. A second method used lentiviral vectors to express miRT sequences (sponges, decoys, antagomirs) to *miR-223* in transduced bone marrow stem and progenitor cells transplanted into mice recipients.^51^ Although both systems inhibited miR activity for their specific miR target, there are limitations to both systems. Furthermore, both methods have dose–response problems and neither have the ability to function as a transgene for developmental studies and long-term knockdown of multiple miR family members. A third method used a transgenic mouse expressing a sponge to bind *miR-183* and these mice demonstrated retina defects.^52^ These methods are critically advancing our knowledge of miR function *in vivo* and provide a wealth of knowledge on miR function.

### New miR inhibitor design and function

Our findings advance these methods by using a novel RNA structure that inhibits miR function *in vivo* and can target multiple miRs with the same seed sequence. The PMIS system is more efficient than the sponge or antagomir sequences. The PMIS was designed independently of the Tough Decoy system^50^ and incorporates a unique RNA secondary structure that facilitates *in vivo* anti-miR function. The PMIS has an anti-miR binding sequence including a four nucleotide bulge region. We demonstrate that both Ago and Dicer bind to the PMIS and we speculate that these interactions stabilize the miR–inhibitor complex. Dicer is not required for RISC loading in mammals but its interaction with the PMIS may facilitate the inhibitor's function.^53^ Previous studies have indicated that overexpression of *shRNAs* and *RNAi* hairpin structures could titrate the miR processing machinery and lead to nonspecific effects and toxicity.^48, 54, 55, 56^ We observe no toxic effects or nonspecific dysregulation of miRs in PMIS-expressing cells or in over 500 mice expressing different miR inhibitors. Each PMIS-miR inhibitor mouse line has specific defects associated with the expression pattern of the specific miR.

**Figure 14:**
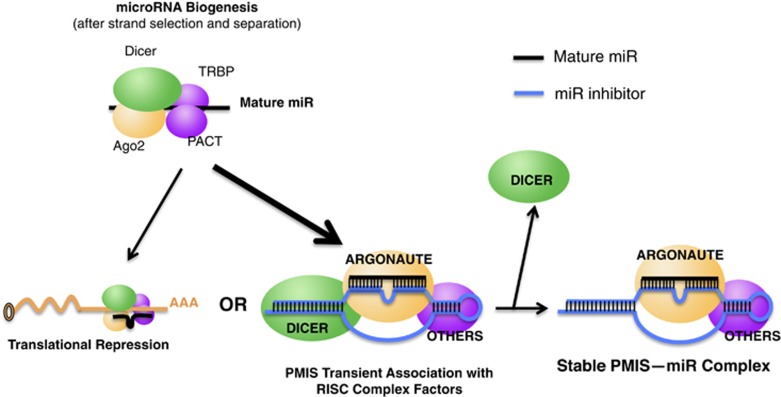
Working model for inhibitor complex. The mature miR is processed and associated with proteins that facilitate its interaction into an RNA-induced silencing complex with the mRNA or the PMIS. The PMIS transiently binds these factors in association with the mature miR. Dicer is associated with AGO2 and TRBP to facilitate transfer of the miR to the PMIS. However, Dicer is released as it cannot cleave the PMIS double-stranded stem. Yellow ellipsoid is AGO protein, green ellipsoid is Dicer protein, and purple ellipsoid is other proteins such as TRBP and PACT. Black line is mature miR, blue line is PMIS-miR inhibitor.

We propose a model for PMIS functional inhibition of miR activity (Figure 14). After miR biogenesis, the mature miR can remain complexed with Dicer, Ago2, TRBP (transactivating response RNA-binding protein) and PACT from the RISC and facilitate mRNA target recognition. Dicer associates with TRBP and Ago2 to facilitate transfer of the miR to the RISC. Dicer and Ago2 and other proteins in the complex facilitate binding of the mature miR to the PMIS in a stable complex. Dicer transiently associates with the PMIS double-stranded stem structure and recycles owing to the short-stem length, which it cannot cleave. The PMIS-miR complex is stable in the cell and leads to degradation of the miR, processed by as of yet an unknown factor.

### Advantages of the PMIS to analyze miR function and identification of developmental defects

We demonstrate an effective and specific system to inhibit miRs and miR clusters in mice using a transgenic approach. Transgenic mice expressing both *PMIS-miR-17-18* and *PMIS-miR-19-92* have a similar phenotype as the *miR-17-92* knockout mice, demonstrating the efficiency and specificity of the PMIS system *in vivo*.^38^ Furthermore, the PMIS can dissect the function of specific miRs in the *miR-17-92* clusters, which is an advantage over traditional genomic knockout approaches. We have demonstrated that *PMIS-miR-19-92* and *PMIS-miR-17-19-92* mice have thyroid developmental defects. These data correlate with report showing that miRs in the *miR-17-92* cluster were overexpressed in thyroid cancer cells compared with primary thyrocytes.^49^ Furthermore, by inhibiting specific miRs in the cluster different effects on growth, apoptosis and cellular senescence were observed in these thyroid cancer cells. By inhibiting the *miR-19-92* family, we show that these miRs are required for thyroid development, and inhibition of the complete family of *miR-17-92* causes more severe defects including bone, cervical and craniofacial defects.

### Utility of the PMIS

Compared with genomic knockouts, sponges and other approaches, making and using the PMIS inhibitors is simple, fast, cost effective and non-toxic. It can be used in viral systems to create stable PMIS-expressing cells and tissues in culture, or in transgenic mice to study miR function. The PMIS as a plasmid can be delivered using nanoparticles, viruses and lipid-based systems to treat cancers with aberrant miR expression or to protect cells and tissues from aberrant gene expression. There is great utility for this system as a therapeutic reagent as the PMIS is extremely specific for each miR. Because this is a plasmid-based miR inhibitor, it is easy and inexpensive to use without reapplication in cells that is required for other modified antisense oligonucleotide systems.

## Materials and methods

### Animals

All animals were housed at the University of Iowa, in the Program of Animal Resources and were handled in accordance with the principles and procedure of the Guide for the Care and Use of Laboratory Animals. All experimental procedures were approved by the University of Iowa IACUC guidelines. *PMIS-miR* DNA was excised from the plasmid and used for pronuclear injection. Donor female mice (FVB/NCr), stud male (FVB/NCr), vasectomized male (ICR) and recipient female (ICR) were used in the experiments. Multiple founders (eight for each construct) were analyzed for transgene expression and crossed to BL6 mice and re-evaluated for expression. Observation of a vaginal plug was counted as embryonic (E) day 0.5, and embryos were collected at E14.5, E16.5, E18.5, P0 and P4. Mice and embryos from WT, *PMIS-miR-17-18*, *PMIS-miR-19-92* and *PMIS-miR-200a* were genotyped from DNA extraction of tail biopsies. We crossed *PMIS-miR-17-18* and *PMIS-miR-19-92* transgenic mice. The resulting mice, *PMIS-miR-17-18-19-92*, inhibit all members of *miR-17-92*, *miR-106a-363* and *miR-106b-25* clusters. Northern blots and quantitative real-time PCR (qPCR) confirmed the expression of the miR inhibitors. Observation of a vaginal plug is counted as E0.5, and embryos and neonates were collected at various time points.

### Construction of miR reporter, expression and inhibitor plasmids

To anneal oligos, oligos were heated at 70 °C for 10 min and then slowly cooled to room temperature. For ligation, annealed oligos were phosphorylated by T4 polynucleotide kinase (NEB) first and then ligated into vectors using the Quick Ligation Kit (Roche, Mannheim, Germany) as per the instructions. To construct miR reporters, one perfect match miR binding site was ligated after the *Renilla* luciferase gene in psiCHECK2 (ck2) vector (Promega dual reporter system, Madison, WI, USA) digested with *Not*I and *Xho*I. For example, to construct the miR reporter for *miR-17*, two short oligos: *miR-17* rf, 5′-TCGAATGACCCTACCTGCACTGTAAGCACTTTGCTCGAGCTGC-3′ and miR-17 rr, 5′-GGCCGCAGCTCGAGCAAAGTGCTTACAGTGCAGGTAGGGTCAT-3′ were annealed and cloned into psiCHECK2 vector digested by *Not*I and *Xho*I. The following oligos were also used: miR-200a rf, 5′-TCGAATGACCACATCGTTACCAGACAGTGTTACTCGAGCTGC-3′ and miR-200a rr, 5′-GGCCGCAGCTCGAGTAACACTGTCTGGTAACGATGTGGTCAT-3′ miR-200b rf, 5′-TCGAATGACCTCATCATTACCAGGCAGTATTACTCGAGCTGC-3′ and miR-200b rr, 5′-GGCCGCAGCTCGAGTAATACTGCCTGGTAATGATGAGGTCAT-3′ miR-200c rf, 5′-TCGAATGACCTCCATCATTACCCGGCAGTATTACTCGAGCTGC-3′ and miR-200c rr, 5′-GGCCGCAGCTCGAGTAATACTGCCGGGTAATGATGGAGGTCAT-3′ miR-141 rf, 5′-TCGAATGACCCCATCTTTACCAGACAGTGTTACTCGAGCTGC-3′ and miR-141 rr, 5′-GGCCGCAGCTCGAGTAACACTGTCTGGTAAAGATGGGGTCAT-3′ miR-429 rf, 5′-TCGAATGACCACGGCATTACCAGACAGTATTACTCGAGCTGC-3′ and miR-429 rr, 5′-GGCCGCAGCTCGAGTAATACTGTCTGGTAATGCCGTGGTCAT-3′. To construct a miR expression plasmid, miR genes were PCR amplified that include ~100 bp upstream and 100 bp downstream sequence flanking the ~80 bp stem loop sequence. The PCR product was ligated into pSilencer 4.1 vector (Ambion, Waltham, MA, USA) digested by *Bam*HI and *Hind*III. To construct different designs of miR inhibitors for miR-17, we annealed and ligated the miR-17 binding site with a central bulge flanked by different sequences into pLL3.7 vector (Addgene, Cambridge, MA, USA) digested with *Hpa*I and *Xho*I. To construct the miR inhibitor clone vector, we replaced the miR-17 binding site with two *Bsm*BI sites in the most effective inhibitor design. *Asc*I and *Pme*I sites were inserted between *Apa*I and *Xba*I sites before the U6 promoter. A *Sma*I site was inserted before *Xho*I after the Pol III terminator. This vector is termed pmiRI for plasmid of miR inhibitor. After digestion by *Bsm*BI, pmiRi can be used to clone different miR inhibitors into it after annealing and ligation of different miR binding sites with a central bulge. To link two different miR inhibitors, one plasmid is digested by *Asc*I and *Sma*I to release a fragment of ~500 bp; this fragment can be ligated into another miR inhibitor digested by *Asc*I and *Pme*I. Plasmid constructs are shown in Figure 1c for cell transductions and Supplementary Figure 5 for transgenic mice. Sponge reporter was made by inserting six tandem binding sites for miR-17 into the psiCHECK2 vector.

### Cell culture, transfection and luciferase assays

HEK 293FT (Invitrogen, Waltham, MA, USA) and MDCK (ATCC, Manassas, VA, USA) cells were cultured in Dulbecco's modified Eagle's medium supplemented with 10% fetal bovine serum and penicillin/streptomycin. HEK 293FT cells are plated the day before transfection. Twenty nanograms of miR reporter and 200 ng of miR inhibitor are co-transfected into each well of 12-well plates using Fugene6 (Roche). For exogenous miR expression, 200 ng miR expression plasmid was transfected into the cells. The Dual-Luciferase Reporter assay (Promega, Madison, WI, USA) was performed 48 h after transfection as per the instructions. siRNA targeting Dicer (5′-AAGGACGGUGUUCUUGGUCAAC-3′ and 5′-CUGCUUGAAGCAGCUCUGGAUC-3′) and GFP (5′-AGGACGACGGCAACUACAAGAC-3′) were *in vitro* transcribed and transfected into cells as described.^57^

### Stability assay of the PMIS constructs

MG-63 cells were transduced with *PMIS-miR-200b* lentivirus, and after 48 h, cells were seeded in 6-well plates at the same density (30–40%) with 0.1% dimethyl sulfoxide in Dulbecco's modified Eagle's medium. After 24 h, cells are treated with actinomycin D at the concentration of 5 ng ml^−1^ (marking the time point as day 0). Cells were harvested at different time points and total RNA was isolated using Qiagen miRNeasy Mini Kit (Qiagen). cDNAs were made by using TaKaRa PrimeScript RT Master Mix Kit (Hilden, Germany) The PCR cycles were as follows: initial denaturation at 95 °C for 1 min, 94 °C for 30 s, annealing at 60 °C for 30 s and extending at 72 °C for 30 s. The PCR primers used were as follows: human *β-actin* primers, 5′-CATGTACGTTGCTATCCAGGC-3′ and 5′-CTCCTTAATGTCACGCACGAT-3′ *PMIS-miR-200b* primers, 5′-CTAATCATCATTACCAATCAGACAGTATTA-3′ and 5′-GTCAGCTCTTAGTATTCATGAGATG-3′. Total RNA from each time point and PCR products were detected in agarose gels. All PCR products were confirmed by sequencing.

### Lentivirus production and transduction

For lentivirus production, 6 -cm dish of HEK 293FT cell were transfected with 2.8 μg of psPAX2, 1.9 μg of pMD2.G and 4.5 μg of miR inhibitor or control plasmid using Fugene HD (Roche). Supernatants were collected and passed through 0.45 μm filter 28 h after transfection. To concentrate virus preparations, 40 ml of supernatant was centrifuged at 26 000 r.p.m. for 2 h at 4 °C. Pellets were resuspended in 100 μl of Dulbecco's modified Eagle's medium at 4 °C overnight. For lentivirus transduction of MDCK cells, 1 × 10^5^ cells were seeded in 6 -cm dish immediately before the addition of 30 μl of concentrated virus. MG-63 cells used for actinomycin D experiments were subcultured and transferred to 6-cm dishes at 30% confluence. Virus was added immediately after plating and cultured for 2 weeks with media change every 2–3 days. Puromycin was added for selection and stable PMIS-miR-17-expressing cells were used to analyze the stability of the PMIS.

### Fluorescence-activated cell sorting

Flow cytometry facility of the University of Iowa preformed cells sorting using the Becton Dickinson Aria II (Franklin Lakes, NJ, USA). Cell suspensions were placed in a 12 × 75 mm^2^ test tube with the final cell concentration between 5 × 10^6^ and 30 × 10^6^ cells per ml. Before sorting, cells are filtered through 70 μm nylon mesh (Falcon 352350, Bedford, MA, USA). Cells are then sorted by GFP and collected at 37 °C into 12 × 75 mm^2^ test tube in Dulbecco's modified Eagle's medium medium with 10% fetal bovine serum. The cells are then cultured.

### RT-PCR, western blot and immunofluorescence

For quantification of mature miRs, Taqman probe (Applied Biosystems) were used as per the instructions. Small RNA U6B served as a control. For quantification of miR primary transcripts, iQ SYBR Green Real-Time PCR Supermix (Bio-Rad, Hercules, CA, USA) was used as per the manufacturer's instructions. β-Actin mRNA served as a control. For western blots, ~20 μg of cell lysates were loaded and ran in sodium dodecyl sulfate-polyacrylamide gel electrophoresis. Proteins were transferred to PVDF filters (Millipore, Darmstadt, Germany), immunoblotted using antibodies and detected by ECL reagents (GE Healthcare, Chicago, IL, USA). The dilution of Dicer antibody (Abcam, Cambridge, MA, USA) was 1:500. AGO2 antibody (Cell Signaling, Danvers, MA, USA) was 1:1000. For immunofluorescence, cells were fixed by cold acetone, blocked with 10% serum of secondary antibody, incubated with primary antibody for 1 h and detected by secondary antibody fluorescence. Cells were counterstained with DAPI (4',6-diamidino-2-phenylindole).

### RT-PCR of mature miRs from E18.5 mouse heads

E18.5 embryos were harvested and heads were used for total RNA isolation. Total RNA including miR from mouse tissues was prepared using the miRNeasy Mini Kit (Qiagen). qPCR analysis of selected miRs was performed using TaqMan miR assay probes (Applied Biosystems), and U6B probe (Applied Biosystems) was used as a reference for normalization. *miR-17-92* expression analyses in mouse tissues was performed as follows: cDNA for mature miR quantification were made using the miScript PCR Starter Kit (Qiagen) and quantitative PCR of mature miRs were carried out using the SYBR Green PCR Kit (Qiagen). Primers for detected mature miRs are available upon request.

### Northern blot assay

Cell miR Northern blot used 5′-end DIG (digoxigenin)-labeled mercury-locked nucleic acid miR detection probes for *miR-17* and *U6* from Exiqon (Woburn, MA, USA) according to the manufacturer's instructions. Briefly, total RNA including miR from cells stably expressing *PMIS vector*, *PMIS-miR-17* or *PMIS-miR-17-18* was extracted. Ten micrograms of total RNA were loaded on a denaturing 12% polyacrylamide gel. After transfer, the membrane was hybridized with 5′-end DIG-labeled mercury-locked nucleic acid miR detection probes.

Tissue miR Northern blot used miR/total RNA isolated from the craniofacial region of P0/P1 pups using the miRNA Easy Kit (Qiagen), heated to 70 °C and run on 12% acrylamide/TBE (Tris/Borate/EDTA)/8 m urea gels. Gels were transferred in TBE, rinsed and placed in UltraHyb buffer (Ambion) at 42 °C. Biotin-end-labeled oligos for each miR were synthesized and purified (IDT, Coralville, IA, USA). Following heat denaturation at 95 °C for 2 min, probes were added to the UltraHyb and incubated overnight at 42 °C. Filters were washed two times in 2xSSC (saline sodium citrate), 0.5% sodium dodecyl sulfate, two times in 0.4xSCC, 0.5% sodium dodecyl sulfate, blocked in 1% nonfat dry milk and incubated with streptavidin-alkaline phosphatase for 2 h at room temperature in PBS-Tween. After four washes with PBS-Tween, cells were incubated in 0.1 m Tris, 0.1 m NaCl (pH 9.5) with BCIP (5-bromo-4-chloro-3-indolyl phosphate) and NBT (nitro blue tetrazolium chloride) (0.4 and 0.2 mg ml^−1^; Roche Dig Nucleic Acid Labeling Kit, Roche) and color reaction was allowed to develop in the dark for 2–8 h.

### Immobilization of RNA on agarose beads and pull-down assay

miR inhibitor RNA with and without flanking double-stranded RNA was synthesized *in vitro* by T7 RNA polymerase and DNA oligonucleotide templates. These RNA were biotinylated using 3′-End Biotinylation Kit (Thermo Scientific, Waltham, MA, USA). Biotinylated RNAs were incubated with streptavidin-conjugated agarose beads for 1 h at 4 °C. HEK 293FT cell were collected and sonicated with protease inhibitor on ice. The cell lysate were precleared with beads only. After preclearing, cell lysates were incubated with RNA immobilized on the streptavidin beads and rotated for 12 h at 4 °C. After washing six times, proteins associated with the beads were released from the beads by boiling. Proteins were analyzed by western blot.

A protocol that was previously reported^58^ was used for PMIS pull down. Briefly, miR inhibitor of *miR-17* with and without flanking double strand was produced by *in vitro* transcription. For AGO2 and DICER pull down, cell extract of 293FT cell stably expressing *PMIS vector*, *PMIS-miR-200c* or *PMIS-miR-17* were incubated with AGO2 (Cell Signaling) and DICER (Abcam) antibodies. RNAs were eluted from the pull-down complex and primers for *PMIS-miR-17* and *U6* were used to carry out RT-PCR.

### mRNA microarray, correlations of mRNA fold change with miR binding site

Total RNA was extracted from 293FT cells stably expressing *PMIS vector* or *PMIS-miR-17*. Human Genome microarray hybridization and scan were performed at the Texas A&M University Genomics Core. Microarray data were analyzed with R. Microarray signal intensity were normalized by global median and log2 transformed. We grouped 22k mRNA (genes) into three groups, 20 K mRNA without *miR-17* binding site (predicted with TargetScan), 2 K mRNA with *miR-17* binding site that have context score larger than −0.3 (a score TargetScan used to measure how likely miR can inhibit target mRNA, the lower the score the more likely), another 277 mRNA with *miR-17* binding site that have context score less than −0.3. Distribution of mRNA fold change (*PMIS-miR-17* vs *PMIS vector*) of these three groups was plotted with R using different colors. One-sided K–S test were used to calculate *P*-value for each pair of comparison between these three groups.

### Skeletal preparations

E18.5 embryos were harvested and placed on ice for 20 min and then scalded the embryos in hot tap water (65 °C) for 30 s. After skin removal, the embryos were fixed in 95% ethanol overnight. Embryos were incubated in Alcian blue solution (0.015% Alcian blue 8GX in a 1:4 mixture of acetic acid and 95% ethanol) and Alizarin red solution (0.005% Alizarin red in 1% KOH) for cartilage and bone staining. Extra tissues were removed in 2% KOH, skeletons were cleared in glycerol-KOH solution (1% KOH, 20% glycerol) and stored in a 1:1 mixture of glycerol and 95% ethanol. Images were captured with a stereo zoom microscope (Nikon SMZ800, Melville, NY, USA). The same magnified images were compared between different genotype of embryos.
